# Anti-BVDV Activity of Traditional Chinese Medicine Monomers Targeting NS5B (RNA-Dependent RNA Polymerase) In Vitro and In Vivo

**DOI:** 10.3390/molecules28083413

**Published:** 2023-04-12

**Authors:** Nannan Chen, Dongjun Jiang, Baihui Shao, Tongtong Bai, Jinwei Chen, Yu Liu, Zecai Zhang, Yulong Zhou, Xue Wang, Zhanbo Zhu

**Affiliations:** 1 College of Animal Science and Veterinary Medicine, Heilongjiang Bayi Agricultural University, Daqing 163319, China; nannanchen@byau.edu.cn (N.C.); shaobaihui1110@163.com (B.S.); tongtongbai@byau.edu.cn (T.B.); c297943673@163.com (J.C.); liuyuyf@163.com (Y.L.); zczhang89@126.com (Z.Z.); zhouyulong1980@163.com (Y.Z.); wangxue1003@byau.edu.cn (X.W.); 2 Key Laboratory of Bovine Disease Control in Northeast China, Ministry of Agriculture and Rural Affairs, Daqing 163319, China; 3 Shandong Collaborative Innovation Center for Diagnosis, Treatment and Behavioral Interventions of Mental Disorders, Institute of Mental Health, Jining Medical University, Jining 272067, China; jiangdj364@nenu.edu.cn; 4 Engineering Research Center for Prevention and Control of Cattle Diseases, Daqing 163319, China

**Keywords:** Chinese medicine, plant compounds, BVDV, antiviral, molecular docking technology, NS5B

## Abstract

Natural products have emerged as “rising stars” for treating viral diseases and useful chemical scaffolds for developing effective therapeutic agents. The nonstructural protein NS5B (RNA-dependent RNA polymerase) of NADL strain BVDV was used as the action target based on a molecular docking technique to screen herbal monomers for anti-BVDV viral activity. The in vivo and in vitro anti-BVDV virus activity studies screened the Chinese herbal monomers with significant anti-BVDV virus effects, and their antiviral mechanisms were initially explored. The molecular docking screening showed that daidzein, curcumin, artemisinine, and apigenin could interact with BVDV-NADL-NS5B with the best binding energy fraction. In vitro and in vivo tests demonstrated that none of the four herbal monomers significantly affected MDBK cell activity. Daidzein and apigenin affected BVDV virus replication mainly in the attachment and internalization phases, artemisinine mainly in the replication phase, and curcumin was active in the attachment, internalization, replication, and release phases. In vivo tests demonstrated that daidzein was the most effective in preventing and protecting BALB/C mice from BVDV infection, and artemisinine was the most effective in treating BVDV infection. This study lays the foundation for developing targeted Chinese pharmaceutical formulations against the BVDV virus.

## 1. Introduction

Bovine viral diarrheal disease (BVD) is an infectious disease having a worldwide distribution, mainly causing diarrhea mucosal disease in cattle, resulting in abortion, stillbirths, and malformations in females, with acute BVDV infection causing immunosuppression [1]. BVDV includes two biotypes, cytopathic (cp) and non-cytopathic (ncp) [2,3]. BVDV belongs to the family Flaviviridae, and other members of the same family with it include hepatitis C virus (HCV), dengue virus (DV), and classical swine fever virus (CSFV) (Table S1). All members of the Flaviviridae family share similarities in virion structure, genome, and replication mechanism, among which the most prominent is BVDV, which has been used as an in vitro model for anti-Flaviviridae drug screening and other drug screening [4]. However, there are no specific medicines or effective control methods for BVDV. Previous studies have identified many synthetic antivirals that are effective against many viral infectious diseases [5], but synthetic antivirals usually show limited efficacy and severe side effects [6]. Therefore, finding a highly effective and non-toxic anti-BVDV virus-targeted medicine is the key to the problem.

In recent years, medicinal plants and their bioactive metabolites have emerged as effective and alternative antiviral active agents [7,8]. RNA-dependent RNA polymerase is crucial in viral RNA synthesis and is one of the most important antiviral drug targets. The nonstructural protein NS5B of BVDV has RNA-dependent RNA polymerase activity and is involved in constituting the replicon of the virus. Numerous studies have shown that some plant metabolites can impede viral replication without affecting host physiology or have limited side effects [9,10]. Flavonoids have important health-protective effects, including anti-inflammatory, anti-cancer, and antiviral properties [11]. There are more than 6000 flavonoids in nature, including isoflavones (daidzein), flavanols (quercetin), and flavins (apigenin) [12,13,14,15,16,17]. Flavonoids, or in combination with other antiviral drugs, can enhance the antiviral effect of drugs [8]. Our previous studies had demonstrated the anti-BVDV viral replication effect of quercetin in the early stages of viral infection, suggesting that flavonoids have an important role in anti-BVDV viruses [18]. Another study found that artemisinine has potential utility in treating human and animal flavivirus infections [19]. Curcumin has various biological functions, not only as an antioxidant and antibacterial compound but also as an antiviral compound that inhibits the replication of many viruses such as hepatitis C virus, SARS-CoV-2 ORF8 (7JTL), COVID-19, Dengue virus, Zika virus, and others [20,21,22,23]. In summary, medicinal plants and their bioactive metabolites have become key research targets for developing antivirals due to their excellent antiviral effects and non-toxic side effects. However, due to their complex and diverse composition, many herbal preparations’ antiviral activities and related mechanisms still need to be clarified.

Starting from the RNA-dependent RNA polymerase NS5B, studying anti-BVDV medicines is promising. Therefore, in this study, the structural information of Chinese medicine monomers (Chinese medicine monomers are the active ingredient in Chinese herbal medicine, also known as TCM monomer compounds) and the nonstructural protein NS5B of NADL strain of BVDV were retrieved through the PubChem compound database and Protein Data Bank. Molecular docking was performed using Autodock and Pymol software (Autodock: La Jolla, CA, USA; Pymol: San Carlos, CA, USA). Through the in vivo and in vitro anti-BVDV virus activity studies, the Chinese herbal monomers with significant anti-BVDV virus effects were screened. Their antiviral mechanisms were initially explored to provide reference data for the future development of anti-BVDV virus-targeted Chinese pharmaceutical preparations.

## 2. Results and Discussion

### 2.1. Molecular Docking Results

NS5B is the most important target in the antiviral activities of some medicines; for example, conserved among the seven identified HCV genotypes, NS5B is considered the most promising target against HCV and has emerged as an important target for the development of antiviral medicines against CSFV infection in the Flaviviridae CSFV replication [24,25]. In recent years, several studies have shown that targeting NS5B has an important role in anti-BVDV therapy, and some studies have also found that the E2 protein of BVDV can also be used as a target for medicine action [26,27,28,29]. To this end, we used the nonstructural protein NS5B (RNA polymerase) of the NADL strain of BVDV as the target for molecular docking.

**Figure 1:**
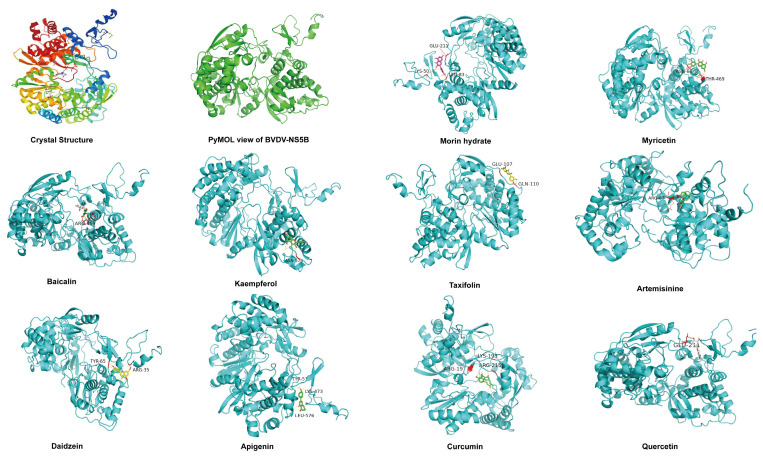
Molecular docking structure diagram.

Ten herbal monomers were individually docked with BVDV-NADL-NS5B via molecular simulation, and the best results were found for artemisinine, daidzein, apigenin, and curcumin. The four herbal monomers’ site-specific molecular docking binding energies were −6.86, −5.64, −5.4, and −4.73 Kcal/mol, respectively (Table 1). The docking structure diagram shows the structure with the lowest docking energy (Figure 1).

### 2.2. Results of Thermal Shift Assay

The principle of Thermal Shift Assay (TSA): Proteins usually exist in a natural state. When the temperature rises, proteins begin to denature, and the hydrophobic region of proteins will be gradually exposed. When the fluorescent dye SYPRO orange is added, it will combine with the hydrophobic protein groups and emit fluorescence. To assess a protein’s thermal stability in different environments, we can determine its melting temperature (Tm). This is the temperature at which 50% of the protein becomes denatured, which can be obtained from the thermal melting curve. A higher Tm indicates better thermal stability for the protein, while a lower Tm means the protein is less thermally stable.

**Figure 2:**
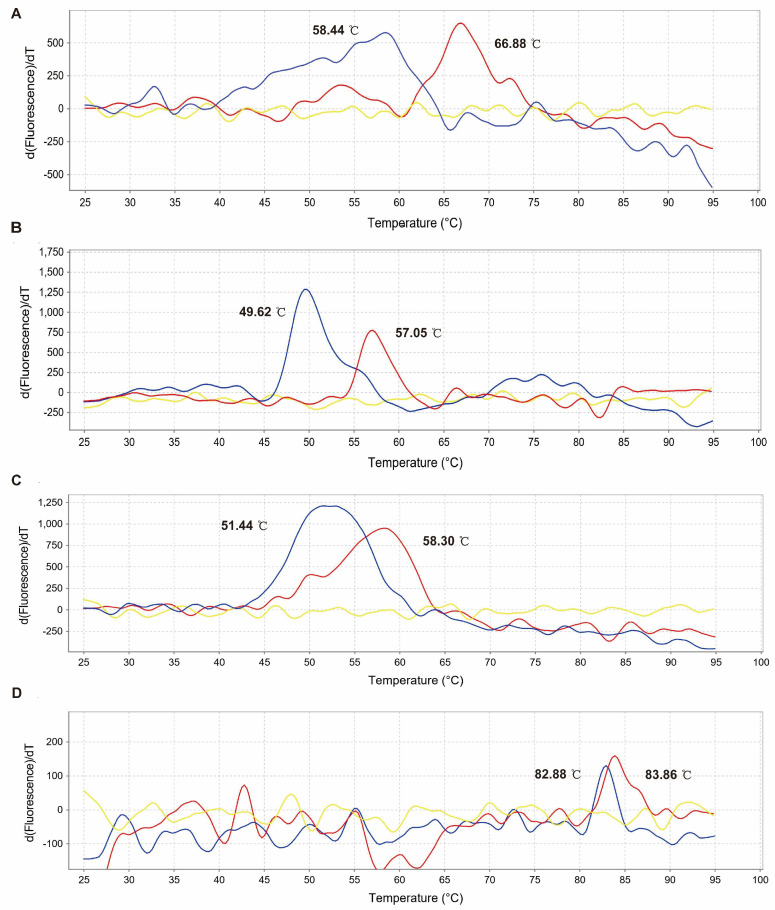
Thermal shift analysis of protein stability and ligand interactions. (**A**): Artemisinine (Tm). (**B**): Daidzein (Tm). (**C**): Curcumin (Tm). (**D**): Apigenin (Tm). The blue curves represent NS5B in the absence of no ligand, the red curves represent NS5B in the presence of Chinese medicine monomer, and the yellow curves are controls.

Melt Curve data generated on a ViiaTM7 Real Time PCR System showing the effect of a specific ligand (Chinese medicine monomer) on protein stability (NS5B). An increase in the protein Tm usually indicates an increase in its stability. We observed a good correlation between NADL-NS5B and small molecules (daidzein, artemisinine, and curcumin). Peptides 1 (artemisinine) showed good TSA stability. Peptides 4 (apigenin) were not stable (Figure 2).

### 2.3. Results of Chinese Medicine Cytotoxicity Assay on MDBK

Flavonoids have a potential role in anti-BVDV viral activity [19,30,31,32]. Flavin-apigenin can inhibit multiple molecular targets of SARS-CoV-2 virus replication [6]. Daidzein also belongs to the flavonoid isoflavones, which can inhibit the growth of leukemia and melanoma cells, and have significant antibacterial effects on *Staphylococcus aureus* and *Escherichia coli* [33]. Studies have reported that artemisinine is active against malaria and has anti-SARS-CoV-2 effects. More importantly, artemisinine significantly reduced the production/release of BVDV viral particles from the embryonic trachea (EBTr) cells infected with the virus. Thus, artemisinine has important applications in treating human and animal viral infections [32,33,34,35]. Curcumin is a pigment extracted from the ginger family member turmeric and is also present in other ginger plants. In addition to having anti-hepatitis C virus, SARS-CoV-2 ORF8 (7JTL), curcumin was found to inhibit CSFV replication in a dose-dependent manner, to inhibit cell binding in Zika virus and also to have antiviral effects against dengue virus [36,37,38]. In summary, Chinese herbal monomers have good antiviral activity with no toxic side effects and are promising Chinese medicinal agents for developing antivirals. However, due to the complexity of the active ingredients of TCM (Traditional Chinese Medicine), their antiviral mechanisms still need to be better understood.

**Figure 3:**
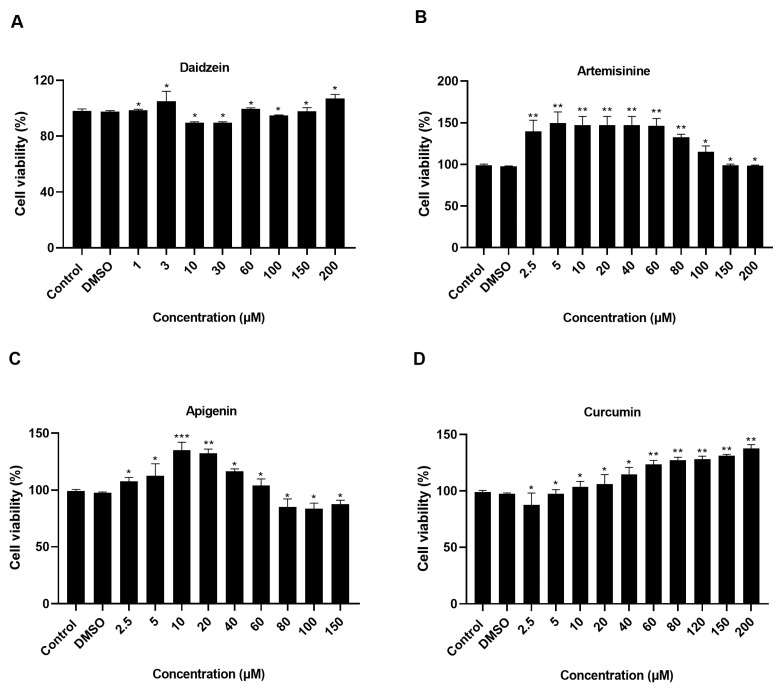
Cytotoxicity results of herbal monomers on MDBK cell. (**A**): Daidzein on MDBK cell viability. (**B**): Artemisinine on MDBK cell viability. (**C**): Apigenin on MDBK cell viability. (**D**): Curcumin on MDBK cell viability. Control: Normal cells. *** *p* < 0.001, ** *p* < 0.01, * *p* < 0.05, *n* = 3. Data were presented as mean ± SD.

Based on the molecular docking results, four herbal monomers (daidzein, artemisinine, apigenin, and curcumin) were selected to study their effects on MDBK cell activity at different doses. It was found that different herbal monomers and concentrations had different effects on MDBK cells. Initially, daidzein led to a rise in the cellular activity of MDBK cells, followed by a subsequent decrease. However, as the concentration of daidzein increased, the cellular activity again increased. Artemisinine and apigenin displayed an initial increase, followed by a subsequent decrease, and curcumin exhibited an increase in a manner that was dependent on the dose administered. It is only at a specific concentration range that the effect of these herbal monomers on cells is minimal, with no major effect on cell survival, and these concentrations are safe for MDBK cells. Therefore, 3 μmol/L of daidzein; 100 μmol/L of artemisinine; 40 μmol/L of apigenin; and 60 μmol/L of curcumin were selected for subsequent experiments (Figure 3).

### 2.4. Results of In Vitro Anti-BVDV Effect of Chinese Medicine on MDBK Cell Activity

To further verify whether each medicine monomer component would cause damage to cells while acting against the BVDV, we examined the effects of different concentrations of herbal monomers on MDBK cells post-infection with BVDV through three different modes of action (post-treatment, pre-treatment, and co-treatment). Then the maximum safe concentration of the four medicines was used as the basis for sequential two-fold dilution into five different concentrations (Daidzein: 3, 1.5, 0.75, 0.375, and 0.187 μmol/L; Artemisinine: 100, 50, 25, 12.5, and 6.25 μmol/L; Apigenin: 40, 20, 10, 5, and 2.5 μmol/L; Curcumin: 60, 30, 15, 7.5, and 3.75 μmol/L).

**Figure 4:**
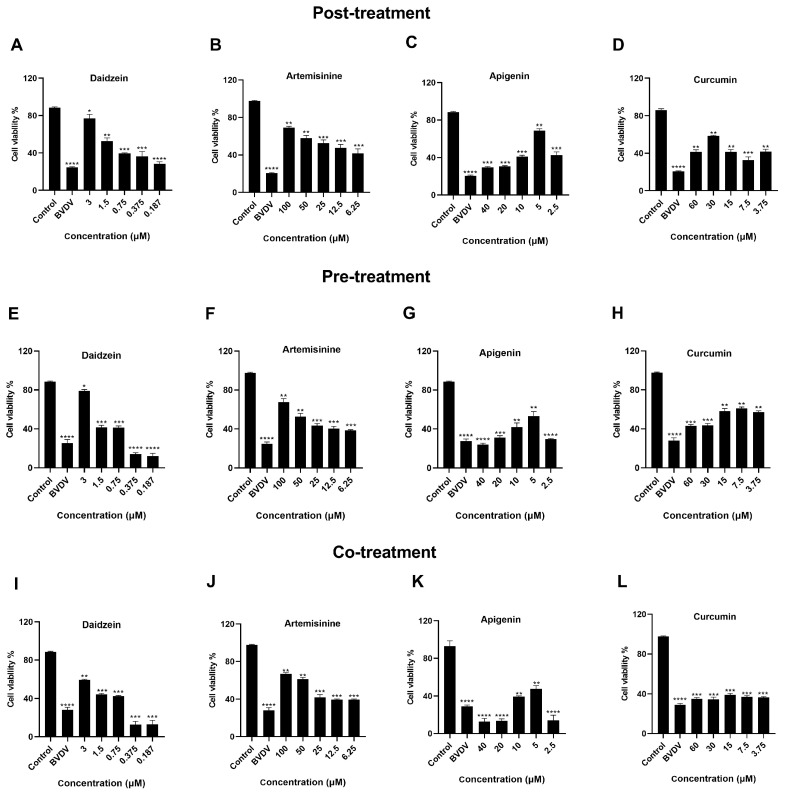
Results of in vitro anti-BVDV effects of Chinese herbal monomers on MDBK cell activity. (**A**–**D**): Effect of Chinese medicine monomeric inhibition of viral infection on MDBK cells. (**E**–**H**): Effect of Chinese medicine monomer to prevent viral infection on MDBK cells. (**I**–**L**): The combined effect of Chinese monomer and virus on MDBK cells. Post-treatment: pre-infection with the virus, then administer the medicines. Pre-treatment: pre-add the medicine, then infect with the virus. Co-treatment: medicine and virus were administered simultaneously. MOI = 1. **** *p* < 0.0001, *** *p* < 0.001, ** *p* < 0.01, * *p* < 0.05, *n* = 3. Data were presented as mean ± SD.

The protection rate of the medicines on MDBK cells infected with BVDV was detected by introducing the virus first and then administering the medicine (post-treatment). The results showed that all four medicines had a good protective effect on MDBK cells post-BVDV infection. Daidzein and artemisinine showed a dose-dependent decrease in cell protection rate with decreasing herbal monomer concentrations. At low concentrations, the drug’s inhibitory effect on BVDV is reduced, affecting cell activity. The protection rate of apigenin and curcumin on cells decreased first, then increased, and finally decreased with the decrease in drug concentration. This indicates that the drug can inhibit the virus at high concentrations but also have certain effects on cells. Therefore, the virus can only be inhibited without affecting cell activity when the drug is at a certain concentration. The concentrations of daidzein, artemisinine, apigenin, and curcumin for the best effective inhibition rate were 3 μmol/L, 100 μmol/L, 5 μmol/L, and 30 μmol/L, respectively (Figure 4A–D).

The medicine was diluted as described above, and its protection rate against BVDV-infected MDBK cells was tested by administering the medicine, followed by introducing the virus (pre-treatment). The results showed that all four medicines proffered better protection to MDBK cells post-BVDV infection. Daidzein, artemisinine, and apigenin results were similar to the post-treatment group. Interestingly, the protective effect of curcumin was observed to be less at high concentrations and higher at low concentrations on cells. This indicates that curcumin has some effects on cell activity at high concentrations. The concentrations of effective prophylaxis in the range of maximum medicine safety concentrations were 3 μmol/L, 100 μmol/L, 5 μmol/L, and 7.5 μmol/L for daidzein, artemisinine, apigenin, and curcumin, respectively, with the most effective medicine for BVDV prophylaxis being daidzein (Figure 4E–H).

The medicine was diluted as described above, and the protection rate against MDBK cells infected with BVDV was tested by simultaneously adding the medicine and virus (co-treatment). The results showed that daidzein and artemisinine had a good protective effect on MDBK cells post-BVDV infection. The curcumin groups did not show a significant difference. In general, adding curcumin and the virus together affected the cells. Apigenin significantly impacted cell activity at both high and low concentrations. The concentrations of the effective rate of daidzein, artemisinine, apigenin, and curcumin in the range of maximum medicine safety concentration were 3 μmol/L, 100 μmol/L, 5 μmol/L, and 15 μmol/L (Figure 4I–L). In summary, daidzein has the most prominent effect in pre-treatment. Artemisinin showed a good effect in pre-treatment, post-treatment, and co-treatment, with the effect of the post-treatment group being slightly more prominent. Curcumin’s effect is better in pre-treatment, and apigenin’s effect is better in the post-treatment group.

### 2.5. Results of Chinese Medicine Monomer on BVDV Virus Replication

The effects of four herbal monomers, daidzein, artemisinine, apigenin, and curcumin, on BVDV virus replication, were examined using three modes of action (introducing the virus first, followed by the administration of the medicine; administering the medicine first, followed by virus introduction, and administering medicine and virus simultaneously).

**Figure 5:**
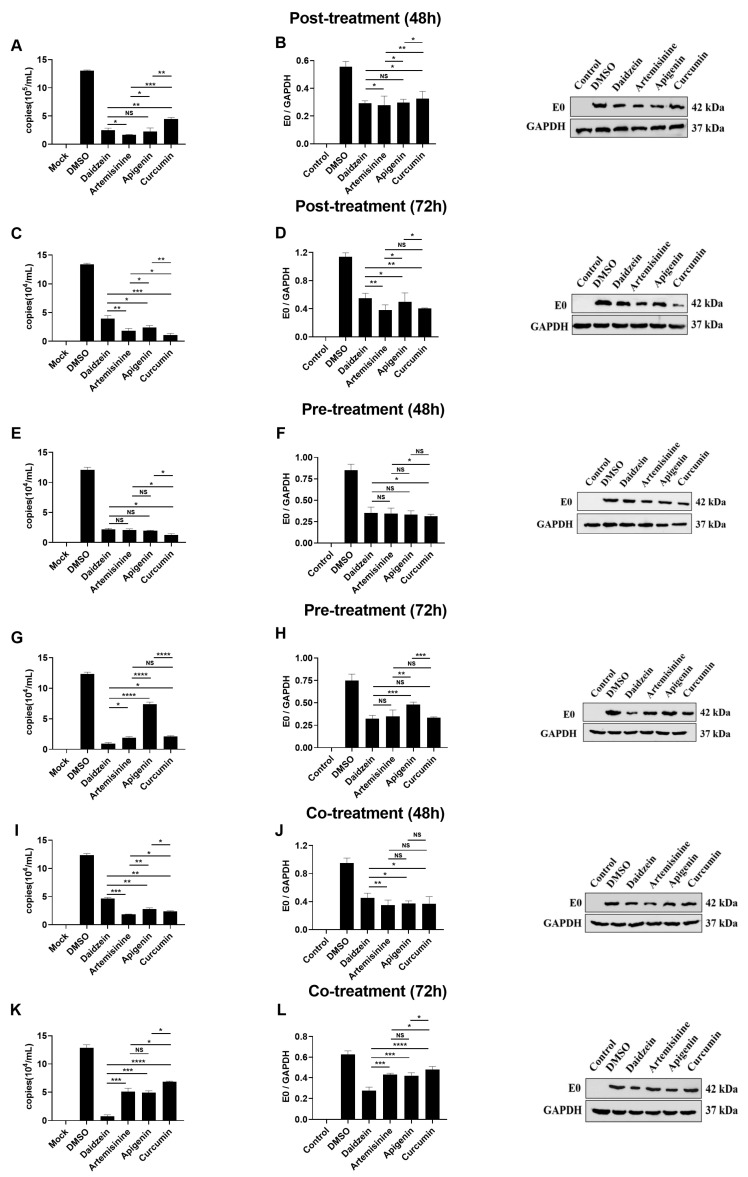
Results of Chinese herbal monomers on BVDV virus replication. (**A**,**B**): Viral mRNA and protein expression levels in MDBK cells after 48 h of BVDV inhibition using Chinese herbal monomers. (**C**,**D**): Viral mRNA and protein expression levels in MDBK cells after 72 h of BVDV inhibition using Chinese herbal monomers. (**E**,**F**): Infected with BVDV after pre-addition of Chinese herbal monomers and detected viral mRNA and protein expression levels in MDBK cells after 48 h. (**G**,**H**): Infected with BVDV after pre-addition of Chinese herbal monomers and detected viral mRNA and protein expression levels in MDBK cells after 72 h. (**I**,**J**): The Chinese herbal monomer and BVDV were co-incubated, and the viral mRNA and protein expression levels were detected in MDBK cells after 48 h. (**K**,**L**): The Chinese herbal monomer and BVDV were co-incubated, and the viral mRNA and protein expression levels were detected in MDBK cells after 72 h. NS: The difference was not significant. **** *p* < 0.0001, *** *p* < 0.001, ** *p* < 0.01, * *p* < 0.05, *n* = 3. Data were presented as mean ± SD.

The virus was first introduced, followed by the administration of the medicine (post-treatment). Then the effect of the four medicines on BVDV virus replication in MDBK cells was examined. The results showed that all four compounds significantly inhibited the viral RNA copy number (*p* < 0.001). Compared with other compounds, artemisinin significantly inhibited viral RNA copy number at 48 h (*p* < 0.05), and curcumin had the most significant inhibitory effect at 72 h (*p* < 0.05) (Figure 5A–D). The effect of the four drugs on preventing the BVDV virus in MDBK cells was examined by administering the drugs first, followed via virus introduction (pre-treatment). The results showed that the four compounds significantly inhibited the copy number of viral RNA (*p* < 0.001). Compared with other compounds, curcumin significantly inhibited viral RNA copy number at 48 h (*p* < 0.05), while daidzein had the most significant inhibitory effect at 72 h (*p* < 0.05) (Figure 5E–H). We examined the killing effect of the four medicines on the BVDV virus in MDBK cells when the medicines and virus were administered simultaneously (co-treatment). The results showed that all four compounds significantly inhibited viral RNA copy number (*p* < 0.001). Compared with other compounds, artemisinine significantly inhibited viral RNA copy number at 48 h (*p* < 0.05), while daidzein had the most significant inhibitory effect at 72 h (*p* < 0.05) (Figure 5I–L).

According to the above results, artemisinine had the best inhibitory effect on virus copy numbers 48 h after post- and co-treatments. On the other hand, daidzein had the strongest inhibitory effect on viral copy number 72 h after pre- and co-treatments. Curcumin was the best at inhibiting viral RNA copy numbers 48 h after pre-treatment and 72 h after post-treatment. Apigenin also greatly inhibited viral RNA/mL copy number, but not significantly compared with other compounds. In conclusion, all four compounds were able to inhibit BVDV viral RNA copy number in MDBK cells significantly and may block or inhibit viral replication during prophase or metaphase stages.

### 2.6. Results of the Effect of Chinese Medicine on the Replication Cycle of the BVDV

The nonstructural protein 5 (NS5B) of BVDV is required for viral replication. NS5B, located at the carboxyl terminus of the polymerase, is highly conserved among pestiviruses and has been proven to have RNA-dependent RNA polymerase (RdRp) activity, which is responsible for the transcription and replication of the viral genome. It has been approved to treat HCV in the US and Europe by targeting NS5B [39]. Because of these unique characteristics, NS5B is an ideal target for developing anti-BVDV antiviral drugs [28]. We have proved that all compounds have a good binding effect with NS5B through molecular docking and TSA experiments. The antiviral activity test showed that all the compounds could significantly inhibit the copy number of viral RNA. Therefore, we speculated that the compound might play an anti-BVDV role by blocking or inhibiting viral replication through interaction with NS5B. To test this hypothesis, we studied the effects of traditional Chinese medicine monomers on the viral replication cycle. We examined BVDV-infected MDBK cells treated with four medicines via inactivation, attachment, internalization, replication, and release patterns using immunoblotting and RT-qPCR.

The results indicate that daidzein primarily affects the early attachment stage of the BVDV replication cycle and has the best preventive effect (pre-treatment) on BVDV replication. Similarly, in the experiment evaluating the effect of daidzein on host cell activity against BVDV, it was found that daidzein was most effective when administered in pre-treatment. This indicates that daidzein plays an antiviral role in the early stage of virus infection by blocking virus attachment to host cells. Artemisinine primarily affects the replication stage of the BVDV replication cycle. In the study of anti-BVDV replication, artemisinine exhibits good efficacy against treating and co-treating viruses (post-treatment and co-treatment), particularly in treating BVDV infection. The experiment on the effect of anti-BVDV on host cell activity revealed that artemisinine had a significant effect in all three treatments—pre-, post-, and co-treatments. The effect of artemisinine in the post-treatment group was slightly more prominent. This suggests that artemisinine may have antiviral effects by reducing the production or release of BVDV virions in BVDV-infected cells. Curcumin was effective in the attachment, internalization, replication, and release phases. Curcumin effectively prevents and treats BVDV infection in pre-treatment and post-treatment stages in the study on anti-BVDV replication. Additionally, curcumin was found to be more effective in pre-treatment in the test on the impact of BVDV on host cell activity. Furthermore, curcumin also has a good binding effect with NS5B. Based on our observations, we believe curcumin may prevent the virus from attaching to the host cell during the early stages of infection until the virus is eventually expelled. Apigenin had a weaker binding effect than the other three TCM monomers, and its effect on the replication cycle of BVDV was mainly in the attachment stage. In contrast, the experiment on the effect of anti-BVDV on host cell activity showed that apigenin was more effective post-treatment. This result suggests that apigenin’s poor binding with NS5B might be the reason for its limited antiviral effect, as opposed to an antiviral effect from binding with NS5B. Further research is needed to study the mechanism behind apigenin’s anti-BVDV effect.

**Figure 6:**
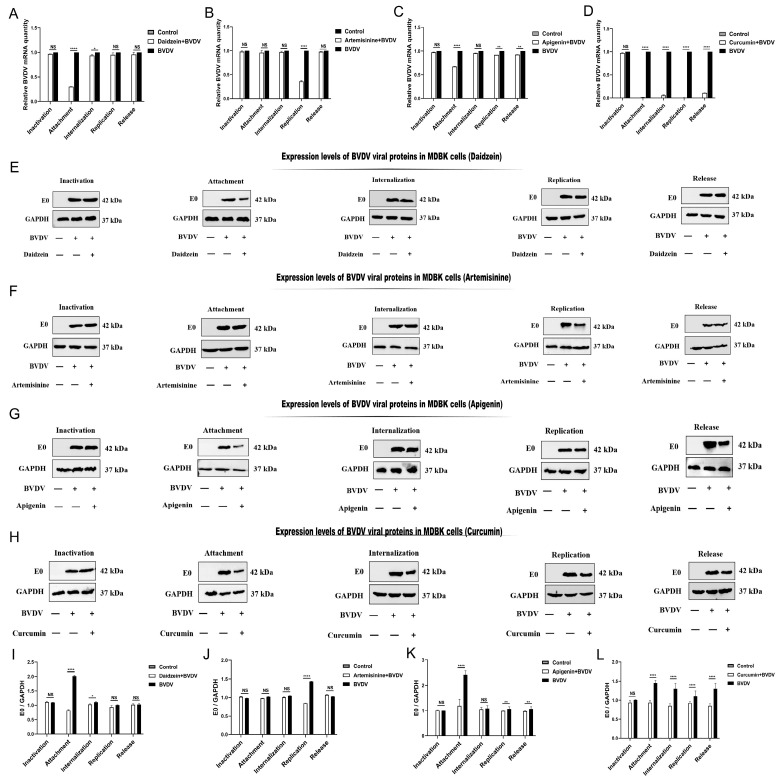
Results of the effect of herbal monomers on the replication cycle of BVDV virus. Daidzein (**A**): BVDV virus mRNA expression level in MDBK cells. (**E**): Expression levels of BVDV viral proteins in MDBK cells. (**I**): Gray value analysis of protein. Artemisinine (**B**): Expression level of BVDV virus mRNA in MDBK cells. (**F**): Expression levels of BVDV viral proteins in MDBK cells. (**J**): Gray value analysis of protein. Apigenin (**C**): Expression level of BVDV virus mRNA in MDBK cells. (**G**): Expression levels of BVDV viral proteins in MDBK cells. (**K**): Gray value analysis of protein. Curcumin (**D**): BVDV virus mRNA expression level in MDBK cells. (**H**): Expression levels of BVDV viral proteins in MDBK cells. (**L**): Gray value analysis of protein. NS: The difference was not significant. **** *p* < 0.0001, ** *p* < 0.01, * *p* < 0.05, *n* = 3. Data were presented as mean ± SD. "—" Indicates: not added. "+" indicates: added.

From the above results, we can see that the effect of TCM monomers on BVDV is mainly in the early stage. Therefore, Chinese medicine monomers can play an anti-BVDV role by preventing the entry of the virus or inhibiting its replication. Importantly, the better the TCM monomer’s molecular docking and TSA binding effect, the better its antiviral activity. For example, molecular docking predicted that artemisinine, daidzein, and curcumin have stronger binding energy. The binding of a ligand to a protein increases protein stability, decreasing the number of hydrophobic groups exposed at the same temperature. A higher temperature is required to expose the hydrophobic groups of the protein fully. Thus, the peptide fragment of NS5B was designed for TSA experiments based on the binding ability and position of NS5B with artemisinin, daidzein, curcumin, and apigenin (determined through molecular docking). The TSA assay results indicated that the protein fragment (NS5B) in complex with artemisinine, daidzein, curcumin, and apigenin had higher thermal stabilization temperatures than the protein fragment alone. This suggests that these compounds have a binding affinity towards BVDV, with artemisinin showing the strongest binding and apigenin showing the weakest binding effect. The antiviral activity results again demonstrated the superior effects of artemisinin, daidzein, and curcumin, while apigenin exhibited anti-BVDV activity but with less stability than the other compounds. This may be attributed to the fact that the better the drug’s binding effect with the protein in the body, the more stable the conformation of the protein will be, leading to a relatively stable antiviral effect. In contrast, due to the weak binding effect between apigenin and NS5B, the anti-BVDV effect may be unstable (Figure 6).

### 2.7. Results of Anti-BVDV Viral Effects of Chinese Herbal Monomers in Mice In Vivo

**Figure 7:**
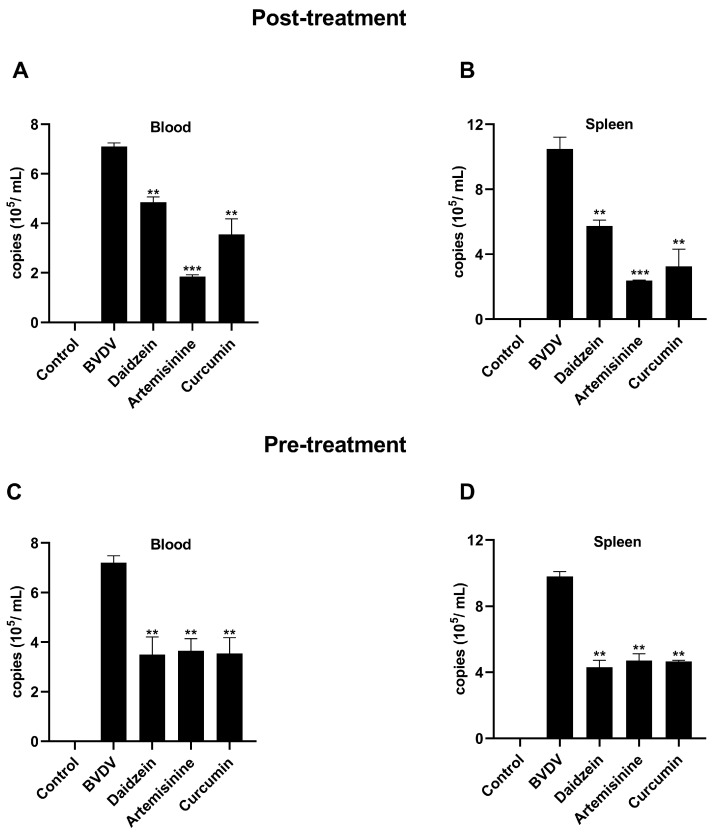
Results of anti-BVDV viral effects of Chinese herbal monomers in mice in vivo. Post-treatment (**A**): Inhibitory effect of Chinese herbal monomers on viruses in blood in vivo. (**B**): Inhibitory effect of Chinese herbal monomers on viruses in splenic in vivo. Pre-treatment (**C**): Effect of Chinese herbal monomers pre-treatment on the viral content of blood in vivo. (**D**): Effect of Chinese herbal monomers pre-treatment on the viral content of splenic in vivo. *** *p* < 0.001, ** *p* < 0.01, *n* = 3. Data were presented as mean ± SD.

To further investigate the effect of herbal monomers on viruses in mice in vivo, daidzein at a concentration of 200 mg/kg, artemisinine at a concentration of 200 mg/kg, and curcumin at a concentration of 150 mg/kg were selected for in vivo antiviral assays. Our previous study in a BVDV mouse infection model found that a high BVDV viral load was detected in the blood and spleen of mice, which are important target organs for viral infection [40]. BVDV viral load was significantly lower (*p* ˃ 0.05) in both the medicine prophylaxis group (medicine administration followed by virus introduction) and the medicine treated group (virus infection followed by medicine administration) compared to the virus control group (Figure 7).

### 2.8. Analysis of Blood Routine Results

**Figure 8:**
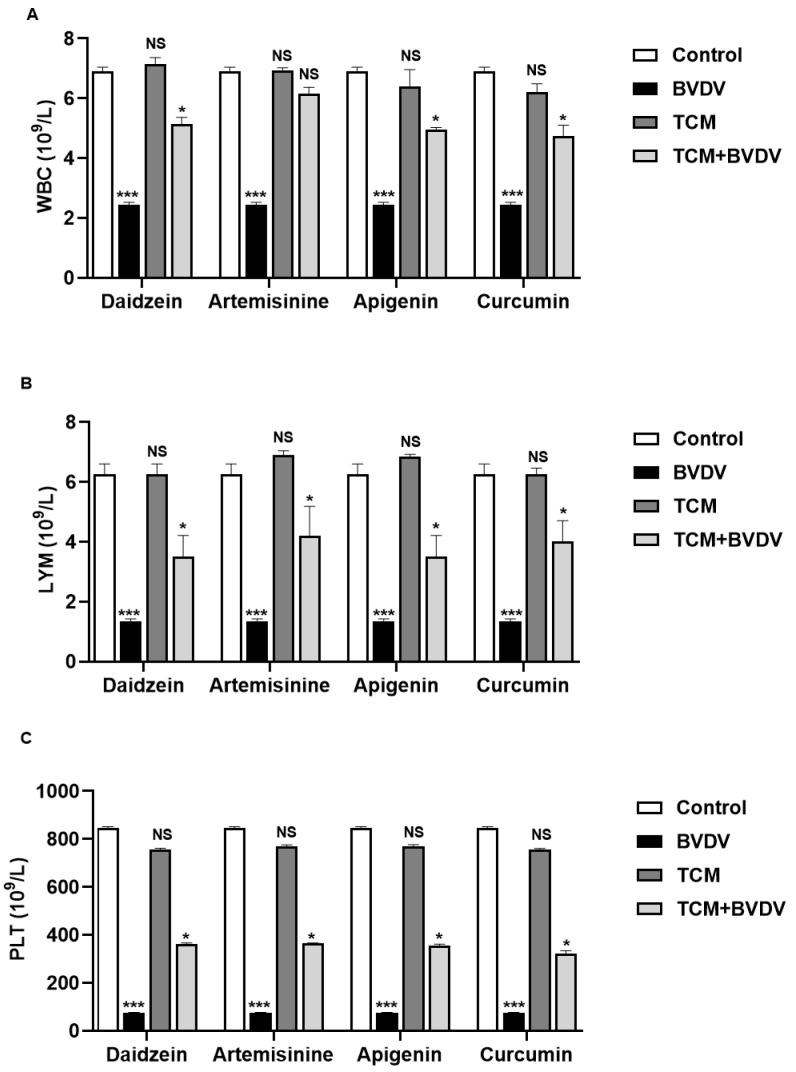
Effect of TCM monomer on the blood indexes of mice. (**A**): White blood cells (WBC). (**B**): Lymphocytes (LYM). (**C**): Platelets (PLT). TCM (Traditional Chinese Medicine). NS: The difference was not significant. *** *p* < 0.001, * *p* < 0.01, *n* = 3. Data were presented as mean ± SD.

The results showed that compared with the blank control group, the values of white blood cells (WBC), lymphocytes (LYM), and platelets (PLT) in mice infected with BVDV decreased significantly (*p* < 0.05). The indexes of four TCM monomers (daidzein, artemisinine, apigenin, and curcumin) were normal. In the drug treatment group, the blood values of WBC, LYM, and PLT of mice treated with TCM monomer were significantly recovered. The therapeutic effect of daidzein and artemisinine was better. In conclusion, the four TCM monomers can help alleviate the decrease of WBC, LYM, and PLT caused by BVDV (Figure 8).

### 2.9. Pathological Histological Analysis Results

According to the different dosing methods and medicine absorption and metabolism mechanisms of the three Chinese medicines, the medicines were absorbed through the intestine after gavage and then metabolized. At the same time, because BVDV infection’s characteristics seriously affect the body’s immune function, the spleen tissue section, the immune organ, was selected. According to the qPCR results, the detection rate of BVDV in blood was similar to that of the spleen, so it was presumed that the spleen might be the main organ for BVDV replication. Two tissues, the spleen and duodenum, were taken for HE staining and observation.

The results showed that BVDV infection caused edema and congestion in the spleen of mice, a significant increase in erythrocytes, a blurred boundary between the red and white marrow, and a significant increase in trabeculae. Medicine-treated BALB/c mice could significantly reduce blood flow in the spleen, increase neutrophils, increase macrophages, and decrease splenic trabeculae. BVDV infection caused small intestinal mucosal congestion, edema, increased inflammatory cells in the submucosa, compensatory widening and shortening of the intestinal villi, decreased crypt foci, and morphological changes. Medicine-treated mice showed a reduction in submucosal inflammatory cell infiltration, an increase in the crypt, an increase in intestinal villi, and an increase in crypt size.

**Figure 9:**
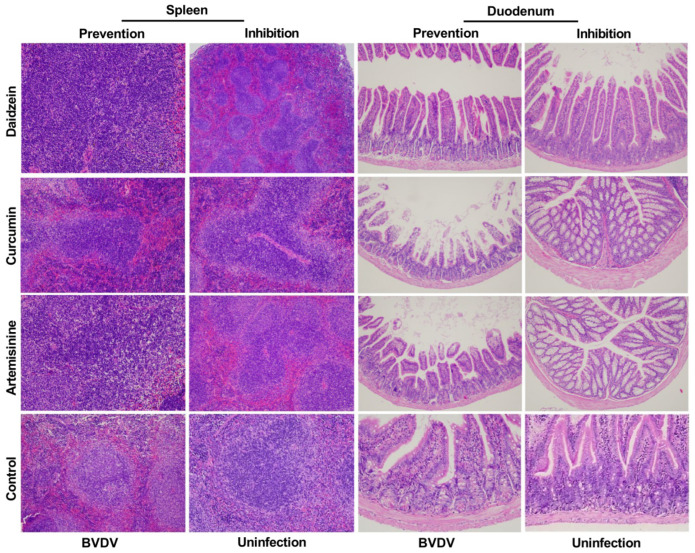
Results of pathological histological analysis. (Hematoxylin and eosin, original magnification, 200×).

The above results showed that all three herbal monomers treated with BALB/c mice could effectively inhibit BVDV infection as well as prevent and protect BALB/c mice. Among them, the more obvious effects were in the daidzein prevention and artemisinine inhibition groups (Figure 9).

## 3. Materials and Methods

### 3.1. Animals and Reagents

BALB/c mice were purchased from Beijing Weitong Lihua Laboratory Animal Technology Co., Ltd. (Beijing, China) (6–8 weeks old, 18–22 g). The cp BVDV-1a (strain NADL, No. VR-534). Daidzein, artemisinine, apigenin, and curcumin were purchased from Chengdu Must Bio-Technology Co., Ltd. (Chengdu, China) (Table S2). and was resuspended in DMSO. CCK8 cell activity assay kit was purchased from Shanghai Biyuntian Biotechnology Co (Shanghai, China).

### 3.2. Autodock, Pymol Molecular Docking

Autodock and Pymol software were used for the molecular docking process, where the Pymol software was used to visualize the 3D structures. Discovery Studio 2016 client software was used to demonstrate 2D and small molecule structures after docking. BVDV-NADL-NS5B (PDBID: 1S48) from Protein Data Bank (http://www.Rcsb.org/pdb, accessed on 1 February 2023) was used for protein crystal structure. Chinese medicine monomer structure was downloaded from the PubChem compound database (https://www.ncbi.nlm.nih.gov/pccompound, accessed on 1 February 2023) (Artemisinine PubChem CID68827; Daidzein PubChem CID5281708; Curcumin PubChem CID969516; Apigenin PubChem CID5280443; Myricetin PubChem CID5281672; Morin hydrate PubChem CID16219651; Kaempferol PubChem CID5280863; Baicalin PubChem CID64982; Quercetin PubChem CID5280343; and Taxifolin PubChem CID439533).

### 3.3. Thermal Shift Assay (TSA)

The specific operational flow of the Thermal Shift Assay (TSA): a. Protein macromolecular preparation: The amino acid sequence of NADL-NS5B protein (abbreviated as “peptide 1–4”) is synthesized, as shown in Table 2. The peptide was diluted into 200 μM protein solution with protein diluent and stored at −20 °C for later use [the peptide diluent was 1 × DPBS (pH 7.2 ± 0.2). DPBS: Dulbecco’s Phosphate Buffered Saline, containing Potassium Chloride (KCl), Potassium Phosphate monobasic (KH_2_PO_4_), Sodium Chloride (NaCl), and Sodium Phosphate dibasic (Na_2_HPO_4_-7H_2_O)]. b. Preparation of small molecules of traditional Chinese medicine monomer ligand: Traditional Chinese medicine monomers (daidzein, artemisinine, apigenin, and curcumin) was diluted with DMSO into 500 mM solution. c. The TSA test reaction system is shown in Table 3. The solution of the above system was added to the 96-well plate and placed on the QuantStudio 3 RT-PCR system. Reaction procedure: 25 °C for 2 min, 25–95 °C with gradual warming (0.05 °C/s), 95 °C for 2 min, and the fluorescence signal was recorded every 20 s. Each sample had 3 multiple Wells. Software: Protein Thermal Shift Software 1.4 was used to analyze the result data.

### 3.4. Cytotoxicity Test

MDBK cells in good growth condition were inoculated in 96-well cell culture plates. DMEM serum-free medium was used to dilute the Chinese medicine monomers to different concentrations (Daidzein: 1, 3, 10, 30, 60, 100, 150, and 200 μmol/L; Artemisinine: 2.5, 5, 10, 20, 40, 60, 80, 100, 150, and 200 μmol/L; Apigenin: 2.5, 5, 10, 20, 40, 60, 80, 100, and 150 μmol/L; Curcumin: 2.5, 5, 10, 20, 40, 60, 80, 120, 150, and 200 μmol/L.), and a DMSO control group was set (the DMSO concentration was 0.1%). Then proceed with the CCK8 cell activity assay kit. The treated cell culture plates were placed in a 5% carbon dioxide incubator, incubated at 37 °C for 48 h, and OD 450 was determined via an enzyme label to calculate the cell survival rate in each group.

### 3.5. Effect of Chinese Medicine Monomers Anti-BVDV on MDBK Cell Activity In Vitro

The assay prepared for adding the medicine examined the rate of protection of MDBK cells infected with the BVDV virus using different concentrations of herbal monomers.

(1) Post-treatment (the virus was introduced first, followed by the administration of the medicine): According to the cytotoxicity assay results of the herbal monomers on MDBK, each herbal monomer was sequentially diluted to 5 concentrations in a twofold gradient with cell maintenance solution, then treated with 0.22 μm filter membrane and prepared for use (Daidzein: 3, 1.5, 0.75, 0.375, 0.187 μmol/L; Artemisinine: 100, 50, 25, 12.5, 6.25 μmol/L; Apigenin: 40, 20, 10, 5, 2.5 μmol/L; Curcumin: 60, 30, 15, 7.5, 3.75 μmol/L). The well-grown MDBK cells were inoculated into 96-well cell culture plates at 1 × 10^5^ mL/well and incubated at 37 °C in a 5% CO_2_ incubator. Then the BVDV virus solution of 0.1 MOI was used to infect MDBK cells for 1 h and placed in the incubator to continue culturing. After 2 h, the solution containing the virus was discarded, and different dilutions of herbal monomer were added. This liquid was discarded after 4 h, and a cell culture medium was added to continue the culture. After five days, the MDBK cell’s survivability was assayed according to the CCK8 kit manufacturer’s instructions. An enzyme marker measured the OD450 to calculate and determine the cell survival rate of each group.

(2) Pre-treatment (the medicine was administered first, followed by the virus introduction) was performed using the above procedure. Different concentrations of herbal monomers were administered first, incubated for 4 h, and discarded (Daidzein: 3, 1.5, 0.75, 0.375, 0.187 μmol/L; Artemisinine: 100, 50, 25, 12.5, 6.25 μmol/L; Apigenin: 40, 20, 10, 5, 2.5 μmol/L; Curcumin: 60, 30, 15, 7.5, 3.75 μmol/L). Then liquid containing BVDV with 0.1 MOI was used to infect MDBK cells and incubated at 37 °C and 5% CO_2_ for 1 h. The viral liquid was discarded after 2 h, and a cell maintenance solution was added to continue the incubation. The cell survival rate of each group was measured after five days.

(3) Co-treatment (medicine and virus were added simultaneously) was performed using the above procedure. MDBK cells were inoculated in 96-well plates, and different concentrations of medicine solution and an equal volume of virus solution were added to each well (Daidzein: 3, 1.5, 0.75, 0.375, 0.187 μmol/L; Artemisinine: 100, 50, 25, 12.5, 6.25 μmol/L; Apigenin: 40, 20, 10, 5, 2.5 μmol/L; Curcumin: 60, 30, 15, 7.5, 3.75 μmol/L). After 4 h of incubation at 37 °C and 5% CO_2_, the liquid was discarded, and cell maintenance solution was added to continue culturing. After five days, each group’s cell survival rate was measured.

### 3.6. Effect of Chinese Medicine Monomers on BVDV Virus Replication

Following the administered experimental medicine method in the previous section, Chinese medicine’s inhibitory effect test (post-treatment), preventive effect test (pre-treatment), and inactivation effect test (co-treatment) were conducted. MDBK cells were inoculated in 24-well cell culture plates, dilutions of different concentrations of Chinese herbal monomers were added (Post-treatment: daidzein: 3 μmol/L; artemisinine: 100 μmol/L; apigenin: 5 μmol/L; curcumin: 30 μmol/L. Pretreatment: daidzein: 3 μmol/L; artemisinine: 100 μmol/L; apigenin: 5 μmol/L; curcumin: 7.5 μmol/L. Co-treatment: daidzein: 3 μmol/L; artemisinine: 100 μmol/L; apigenin: 5 μmol/L; curcumin: 15 μmol/L), and the cells were infected with 1 MOI of BVDV virus solution incubated at 37 °C in a 5% CO_2_ incubator. Samples were collected for RNA and protein isolations at 48 h and 72 h, respectively, for RT-qPCR and Western blotting assays.

### 3.7. Effect of Chinese Medicine on the Replication Cycle of the BVDV Virus [41,42]

To conduct the viral replication cycle test, we selected the compound concentrations based on the results of BVDV replication. Specifically, we used daidzein at 3 μmol/L, artemisinine at 100 μmol/L, apigenin at 5 μmol/L, and curcumin at 7.5 μmol/L.

Inactivation: Equal volumes of Chinese herbal monomer and BVDV (MOI = 1) were incubated at 37 °C for 2 h, and the control was DMSO. The mixture was washed with PBS and ultra-centrifuged at 90,000× *g* for 1.5 h in 20% sucrose buffer (*w*/*w*) to purify the virus. The virus pellet was resuspended in the medium, and the cells were incubated at 37 °C for 2 h. After being washed in PBS, cell lysates were collected for RT-qPCR and Western blotting assays.

Attachment: MDBK cells were pretreated for 1 h using each herbal monomer separately and then incubated with BVDV (MOI = 1) at 4 °C for 2 h. After washing with PBS three times, the cell lysates were collected for RT-qPCR and Western blotting assay.

Internalization: MDBK cells were infected with BVDV (MOI = 1) to allow viral attachment and incubated at 4 °C for 1 h. The cells were washed with PBS three times to remove unbound viral particles. The medium was replaced with another containing DMEM/F-12, FBS (2%), and herbal monomer, which was incubated for 1 h at 37 °C. Cells were washed with PBS to remove the uninternalized virus, and the control was DMSO. Cell lysates were collected for RT-qPCR and a western blotting assay.

Replication: MDBK cells were infected with BVDV (MOI = 1) at 37 °C for 1 h. Cells were washed three times with PBS to remove the non-invasive virus, followed by adding DMEM/F-12 to remove residual PBS. Then DMEM/F-12 and FBS (2%) containing Chinese medicine monomer or DMSO were added to MDBK cells and incubated at 37 °C for 2 h. Cell lysates were collected for RT-qPCR and western blotting assay.

Release: MDBK cells were infected with BVDV (MOI = 1) at 37 °C for 1 h. After washing three times with PBS, the medium was replaced with fresh DMEM/F-12 and FBS (2%). The cells were washed three times with PBS 10 h later, and the medium was replaced with DMEM/F-12 and FBS (2%) containing Chinese medicine or DMSO. Cultures were incubated at 37 °C for 2 h. The supernatant was collected and detected by RT-qPCR and western blotting. Each sample was tested in triplicate, and fold differences in gene expression were calculated using the 2^−ΔΔCt^ method with normalization to β-actin.

### 3.8. Anti-BVDV Viral Effect of Chinese Herbal Monomers in Mice In Vivo

BALB/C mice were divided into five groups: virus control, healthy control, medicine control (DMSO), medicine prevention (pre-treatment), and medicine inhibition (post-treatment). Each group had six mice. The mice were administered 200 μL of the medicines via gavage. To infect the mice, 0.4 mL of BVDV with a 0.1 MOI value was intraperitoneally injected, following our previously established animal model method [37].

The Chinese medicine monomers prophylaxis group was administered via gavage for three days before intraperitoneal injection with 0.4 mL of BVDV viral solution at 1 MOI. The medicine was then administered via gavage for seven consecutive days. For the medicine-inhibited group, the mice were administered the medicine via gavage for seven consecutive days after the first day of BVDV intraperitoneal injection. The healthy control group was administered saline via gavage, while the medicine control group was administered DMSO. All mice were sacrificed after seven days, and organ and blood samples were collected for analysis.

### 3.9. Routine Blood Test

To identify the influence of traditional Chinese medicine monomers on the blood indexes of mice, a routine blood test of mice was performed. The experiment was divided into blank control, TCM monomer, and drug treatment groups. According to the above animal test method, on the 7th day of virus infection, the blood of mice was collected and analyzed using the whole blood cell analyzer.

### 3.10. Histopathological Analysis

Portions of the duodenum and spleen of mice in all experimental groups were fixed in 4% paraformaldehyde. The tissue sections were prepared by washing, dehydration, transparency, waxing, embedding, sectioning, spreading, hematoxylin-eosin (H&E) staining, and sealing. The histopathological changes of the duodenum and spleen were examined with a microscope (Ezhou, China).

### 3.11. Statistical Analysis

Statistical analysis was performed using GraphPad Prism version 8.0 (GraphPad software). All data were expressed as mean ± SD. *p* < 0.05 indicated a statistically significant difference. All samples were assayed in triplicate.

## 4. Conclusions

In summary, our results demonstrate that daidzein, artemisinine, and curcumin have potential applications in preventing and controlling BVDV virus infection. However, in-depth research is still needed to study the interaction mechanism between herbal monomers and viruses. The role and mechanism of more herbal medicines in anti-BVDV replication still need to be determined. This study lays a certain foundation for the future development of herbal medicines against Flaviviridae viruses and explores the mechanism of action of herbal preparations.

## Figures and Tables

**Table 1:** Binding energy statistics of Chinese monomers with BVDV-NADL-NS5B.

Name	Binding Energy (kcal/mol)	Order of Scores
Artemisinine	−6.68	1
Daidzein	−5.64	2
Curcumin	−5.43	3
Apigenin	−4.73	4
Myricetin	−4.66	5
Morin hydrate	−4.65	6
Kaempferol	−4.45	7
Baicalin	−4.41	8
Quercetin	−4.4	9
Taxifolin	−4.29	10

**Table 2:** The amino acid sequence of NADL-NS5B protein.

Peptide	PubChem CID	Sequence
Peptides 1	Artemisinine PubChem CID68827	(164–175) TFHEAIRDKIDK
Peptides 2	Daidzein PubChem CID5281708	(265–273) EKRDVSDDW
Peptides 3	Curcumin PubChem CID969516	(313–323) IPGYEGKTPLF
Peptides 4	Apigenin PubChem CID5280443	(395–406) EVYIRNGQRGSG

**Table 3:** The TSA test reaction system.

Sample	Volume (μL)
Peptide (200 μM)	2
Chinese medicine monomer 500 mM	2
SYBR range (8×)	5
ddH_2_O	11
total	20

## Data Availability

The raw data supporting the conclusions of this article will be made available by the authors without undue reservation.

## Supplementary Materials

The following supporting information can be downloaded at: https://www.mdpi.com/article/10.3390/molecules28083413/s1, Table S1: Abbreviations list; Table S2: Compound structural formula.

## Sample Availability

Not available.
