# Supplementary material for: Anti-BVDV Activity of Traditional Chinese Medicine Monomers Targeting NS5B (RNA-Dependent RNA Polymerase) In Vitro and In Vivo
Source: Molecules. 2023 Apr 12;28(8):3413. doi: 10.3390/molecules28083413 (PMC10145726; doi:10.3390/molecules28083413)
Supplement: Supplementary file 1 [file molecules-28-03413-s001.zip › molecules-2242015-supplementary.pdf]

Supplementary data

Table S1. Abbreviations list.

Abbreviations	Full name of the acronym
BVDV	Bovine viral diarrhea virus
HCV	Hepatitis C virus
DV	Dengue virus
CSFV	Classical swine fever virus
HBV	Hepatitis B Virus
PEDV	Porcine epidemic diarrhea virus
EBTr	Embryonic trachea
TCM	Traditional Chinese Medicine
WB	Western blotting
qRT-PCR	Quantitative Real-time PCR
H&E	Hematoxylin-eosin

Table S2. Compound structural formula.

Compound structural formula	Name	Abbreviations	CAS No.
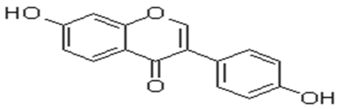	Daidzein	C15H10O4	486-66-8
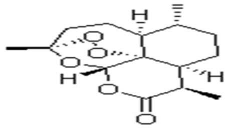	Artemisinine	C15H22O5	63968-64-9
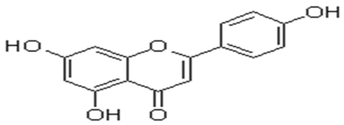	Apigenin	C15H10O5	520-36-5
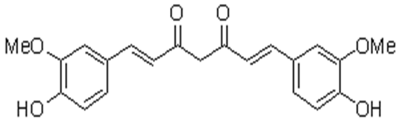	Curcumin	C21H20O6	458-37-7
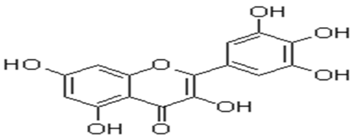	Myricetin	C15H10O8	529-44-2

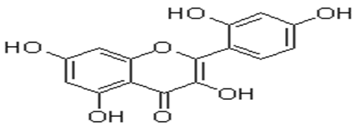	Morin hydrate	C15H10O7	480-16-0
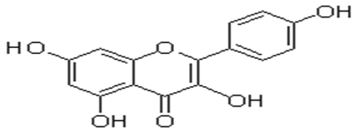	Kaempferol	C15H10O6	520-18-3
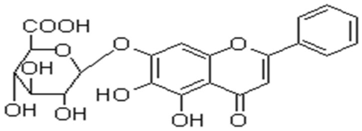	Baicalin	C21H18O11	21967-41-9
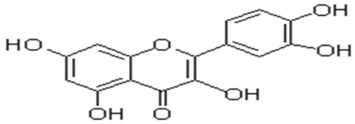	Quercetin	C15H10O7	117-39-5
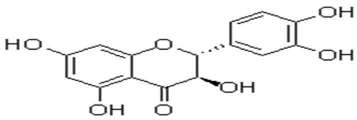	Taxifolin	C15H12O7	480-18-2
